# EPDR1 is related to stages and metastasize in bladder cancer and can be used as a prognostic biomarker

**DOI:** 10.1186/s12894-021-00843-2

**Published:** 2021-04-26

**Authors:** Yue Yang, Hanchao Zhang, Zhengdao Liu, Faliang Zhao, Guobiao Liang

**Affiliations:** 1 Medical College of Soochow University, Suzhou, Jiangsu China; 2 Urological Department, The Affiliated Hospital and Clinical Medical College of Chengdu University, Chengdu, Sichuan China; 3 Urological Department, The Affiliated Hospital of Zunyi Medical University, Zunyi, Guizhou, China

**Keywords:** Biomarker, EPDR1, Bladder cancer, Immune cell infiltration

## Abstract

**Background:**

Bladder cancer (BLCA) is a malignant urothelial carcinoma and has a high mortality rate. EPDR1 (ependymin related 1) is a type II transmembrane protein and related to calcium-dependent cell adhesion.

**Methods:**

We explored the potential oncogenic roles of EPDR1 in BLCA basing on the multiple public datasets.

**Results:**

We found that EPDR1 expression had a significant difference in BLCA and adjacent normal bladder tissues, and the level of EPDR1was up-regulated with advanced tumor stage and metastasis in BLCA. Meanwhile, the high expression group of EPDR1 had a shorter OS compared to the low or medium expression-group. Furthermore, EPDR1 expression was associated with tumor-infiltrating immune cells (TIICs), including NK cells, CD8 + T cells, CD4 + T cells, Macrophages cells, and so on. Moreover, EPDR1 also involved in several signaling pathways as well as PI3K/AKT pathway, Cytokine receptor interaction, and apoptosis.

**Conclusion:**

EPDR1 can be used as a novel prognostic biomarker as well as an effective target for diagnosis and treatment in BLCA.

## Introduction

Bladder cancer (BLCA) accounts for an estimated 500,000 new cases and 200,000 deaths worldwide, and it is the fourth most common malignancy among men in the western countries, which is 3 to 4 times more common than in women [1, 2]. However, treatment for BLCA had seen little progress until recently, thus there is a lot to do. At one end of the spectrum, low-grade BLCA would have a low progression rate and a threat to the patient; at the other extreme, high-grade BLCA has a high malignant potential associated with significant progression and cancer death rates [3]. Attempting to predict tumor behavior and prognosis, it is important to explore many characteristics, including the molecular markers and it will help in the prevention of BLCA progress.

The ependymin-related 1 (EPDR1) gene, also known as MERP1 and UCC1, encodes for a protein related to ependymins, which are type II transmembrane proteins that are similar to two families of cell adhesion molecules: the protocadherins and ependymins [4]. In 2001, EPDR1 was firstly reported and designated as UCC1 by Nimmrich et al. [5] in two colorectal cancer (CRC) cell lines, which up-regulated in colon cancer and displayed some similarity to the ependymin genes. Afterward, Kirkland et al. [6] also found a gene highly expressed in hematopoietic cells and several malignant tissues and cell lines, which was named MERP1 and turned out to be the same as UCC1. Lately, several studies [7–9] were reported that EPDR1 was closely related to pathological or developmental processes of various tumors including breast cancer (BRCA) [10], hepatocellular carcinoma (HCC) [11], colorectal cancer, and so on.

However, the function of EPDR1 was never reported in BLCA. In our current study, we used several databases to intend to explore the correlation and mechanism of EPDR1 in tumorigenicity and metastasis of BLCA, and we employed Univariate Cox analysis to construct a more accurate prognostic signature.

## Materials and methods

### EPDR1 expression

We investigated the expression of EPDR1 between tumor and adjacent normal tissues in BLCA by TIMER2 (tumor immune estimation resource, version 2) web and Gene Expression Profiling Interactive Analysis (GEPIA) database from the Cancer Genome Atlas (TCGA) database and the GTEx (Genotype-Tissue Expression) database. “Gene_DE” module of TIMER2 and the “Expression analysis-Box Plots” module of the GEPIA were used to explore expression levels of the EPDR1 gene, with the settings of p-value < 0.05, log2FC (fold change) < 1, and “Match TCGA normal and GTEx data”. Additionally, through the “Pathological Stage Plot” module of HEPIA2, different pathological stages (stage II, III, and IV) which expressed various levels of EPDR1 in BLCA were also conducted from the TCGA database. The box or violin plots are applied with log2 [TPM (Transcripts per million) + 1] transformed expression data.

### Immune infiltration analysis

The tumor patients in the TCGA database, tumor RNA-seq data (TCGA), and 408 BLCA patients can be downloaded from the Genomic Data Commons (GDC) data portal website. Each tumor has mRNA expression data from a matched normal tissue sample. To make reliable immune infiltration estimations, we utilize the “immune decode”, an R package that integrates six state-of-the-art algorithms, including TIMER, xCell, MCP-counter, CIBERSORT, EPIC, and quanTIseq. All the above analysis methods and R package were implemented by R foundation for statistical computing (2020) version 4.0.3 and software packages ggplot2 and heatmap.

### Survival analysis

Raw counts of RNA-sequencing data and corresponding clinical information from BLCA were obtained from the TCGA dataset in January 2020, in which the method of acquisition and application complied with the guidelines and policies. The KM survival analysis with the log-rank test was also used to compare the survival difference between the above two groups. For Kaplan–Meier curves, Overall Survival (OS) was applied where p-values and hazard ratio (HR) with 95% confidence interval (CI) were generated by log-rank tests and univariate Cox proportional hazards regression. All analytical methods above and R packages were performed using R software version v4.0.3 (The R Foundation for Statistical Computing, 2020).

### Prognostic significance of EPDR1 expression in BLCA

Univariate and multivariate cox regression analyses were performed to identify the proper terms to build the nomogram. The forest was used to show the p-value, HR, and 95% CI of each variable through the “forest plot” R package. A nomogram was developed based on the results of multivariate Cox proportional hazards analysis to predict the 1 and 3-year overall recurrence. The nomogram provided a graphical representation of the factors, which can be used to calculate the risk of recurrence for an individual patient by the points associated with each risk factor through the “RMS” R package.

### EPDR1-related gene enrichment analysis

We explored the STRING website setting the query of a single protein name (“EPDR1”), organism (“Homo sapiens”), the following main parameters: minimum required interaction score [“Low confidence (0.150)”], meaning of network edges (“evidence”), max number of interactors to show (“no more than 50 interactors”) and active interaction sources (“experiments”). Then, the available experimentally determined EPDR1-binding proteins were obtained. Meanwhile, the “Similar Gene Detection” module of GEPIA2 was conducted to obtain the top 20 EPDR1-correlated targeting genes based on the datasets of all TCGA tumor and GTEx normal tissues. Moreover, we combined the above data to perform KEGG (Kyoto encyclopedia of genes and genomes) pathway analysis. In brief, we uploaded the gene lists to DAVID (Database for annotation, visualization, and integrated discovery) and set of selected identifiers (“OFFICIAL_GENE_SYMBOL”) and species (“Homo sapiens”) and obtained the data of the functional annotation chart. The enriched pathways were finally visualized with the R packages of “tidy” and “ggplot2”. The R language software [R-3.6.3,64-bit] was used in this analysis.

### Tumor mutation burden (TMB)/microsatellite instability (MSI) analysis

The dataset used comprised mRNA-seq data from TCGA Data. TMB is derived from the article The Immune Landscape of Cancer published by Vesteinn Thorsson et al. [12] in 2019; MSI is derived from the Landscape of Microsatellite Instability Across 39 Cancer Types article published by Russell Bonneville et al. [13] in 2017. We used Spearman’s correlation analysis to describe the correlation with EPDR1 between quantitative variables without normal distribution.

### EPDR1 protein levels in BLCA

We explored the expression of EPDR1 protein and path in BLCA on the immunohistochemistry (IHC) data from the Human Protein Atlas (HPA) database (https://www.proteinatlas.org/).

## Results

### EPDR1 expression is significantly different in BLCA tissues

**Fig. 1:**
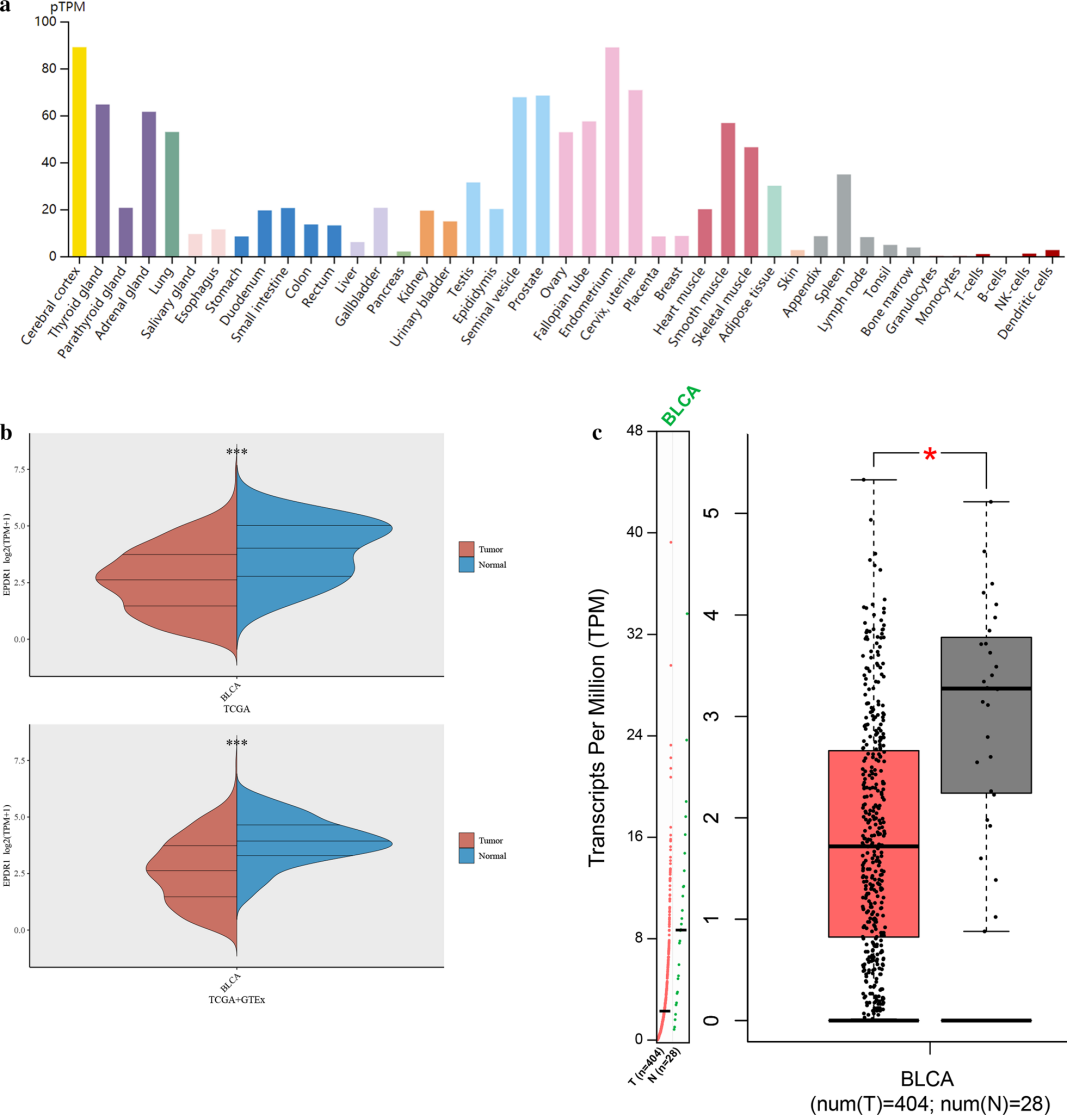
EPDR1 expression. **a** Expression level of EPDR1 in normal human tissues based on the GTEx data. **b** Wilcox test was used for the EPDR1 expression distribution in BLCA and adjacent normal bladder tissues from TCGA or normal bladder tissues from GTEx; **c** Expression on Box Plots of EPDR1 in BLCA from GEPIA. The expression data are first log2(TPM + 1) transformed for differential analysis and the log2FC is defined as median (Tumor) – median (Normal)

Analyzing multiple data from TCGA, GTEx, and GEPIA databases, we found that the expression of EPDR1 was highly expressed in the adrenal gland, blood vessel, lung, nerve tissues, and others (Fig. 1a). However, it had a significantly different expression in BLCA and adjacent normal bladder tissues (Fig. 1b, *p* < 0.001). The expression on Box Plots of EPDR1 in BLCA was conducted from GEPIA and the expression data are first log2(TPM + 1) transformed for differential analysis where the log2FC is defined as median (Tumor) – median (Normal). The results also proved that EPDR1 had a significantly different expression level in BLCA and adjacent normal tissues (Fig. 1c, *p* < 0.05).

### EPDR1 expression related with stages and metastasize

**Fig. 2:**
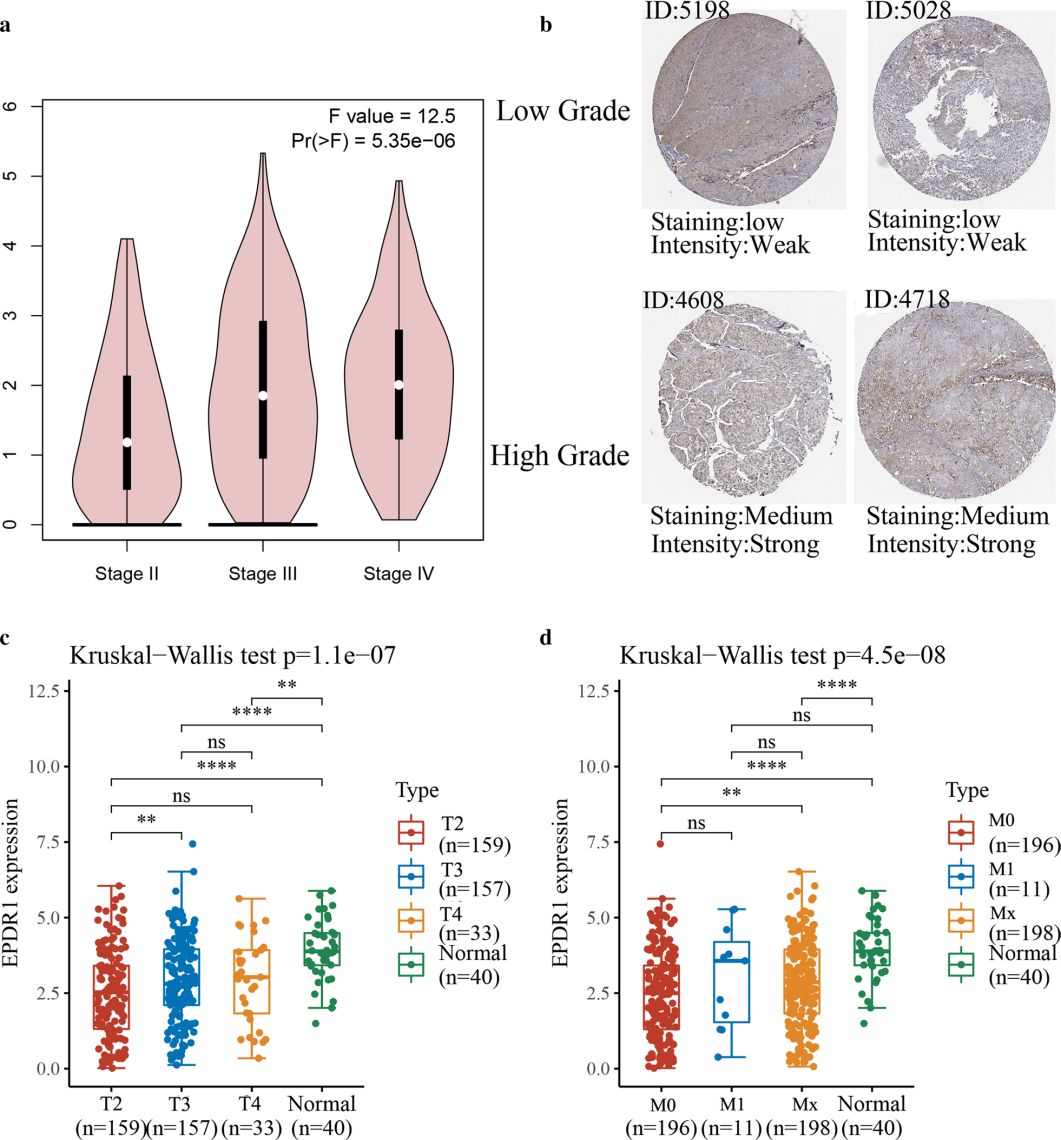
EPDR1 expression related with stages and metastasize. **a** EPDR1 expression analysis in pathological stage by one-way ANOVA from GEPIA databases; **b** The EPDR1 expression levels of high grades or low grade in BLCA from HPA database and CPTAC database; **c** Kruskal–Wallis Test for EPDR1 expression in each clinical tumor stage (T2, T3, T4) and adjacent normal tissues, **d** Kruskal–Wallis Test for EPDR1 expression in metastasis (M0, M1, Mx) and adjacent normal tissues

We explored the correlation of the level of EPDR1 with pathological stages and metastasize in BLCA, and the results revealed that EPDR1 expression was significantly positively related with pathological stages and metastasize from TCGA databases. First of all, basing on the patient pathological stage, EPDR1 expression analysis was conducted by one-way ANOVA from GEPIA databases, using the pathological stage as a variable for calculating differential expression and first log2(TPM + 1) transformed for differential analysis. The results presented that it has significant difference among stages II, III, and IV (Fig. 2a, F = 12.5, *p* < 0.001). Furthermore, we verified the protein levels of EPDR1 in BLCA tissues using HPA and CPTAC databases. We found that EPDR1 was significantly higher in a higher grade of BLCA tissue with strong intensity and medium staining than low grade with weak intensity and low staining (Fig. 2b). Meanwhile, Kruskal–Wallis Test for each tumor stage (T2, T3, T4), metastasis (M0, M1, Mx), and normal tissues also proceeded from TCGA and GTEx databases. The result also showed us that EPDR1 expression was obviously correlated with stages (Fig. 2c, *p* < 0.001) and metastasis (Fig. 2d, *p* < 0.001), thus patients with higher stages or more metastasize would have an increased expression in EPDR1 (Fig. 2d, *p* < 0.01).

### Prognostic significance of EPDR1 expression in BLCA

**Fig. 3:**
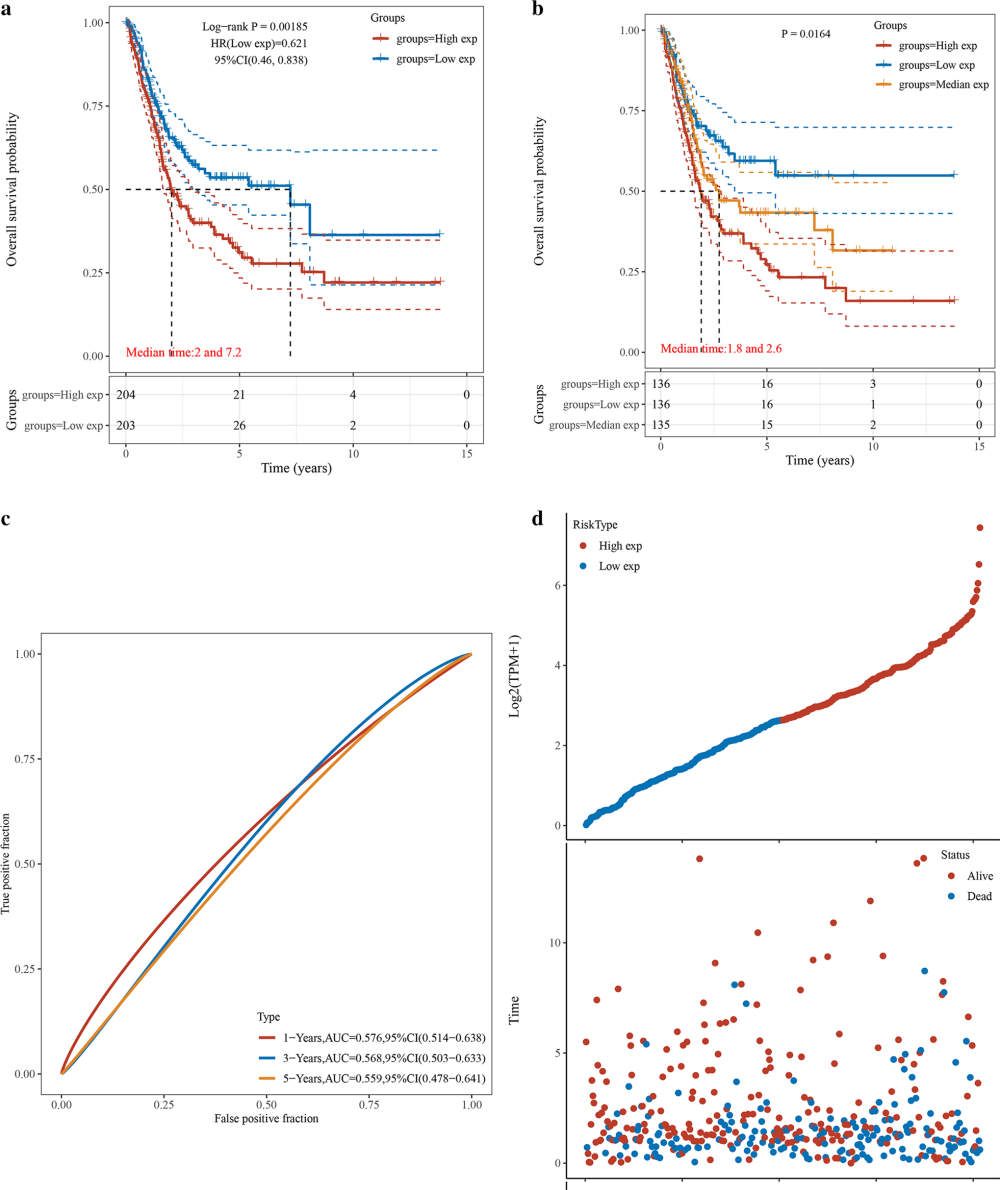
Construction for EPDR1 signature. **a** Kaplan–Meier survival curves were plotted to estimate the overall survival probabilities for the low-risk versus the high-risk group of EPDR1; **b** Kaplan–Meier survival curves were plotted to estimate the overall survival probabilities for the low-risk versus medium group versus the high-risk group of EPDR1; **c** ROC curve was plotted for 1-, 3- and 5-y overall survival; **d** The EPDR1 signature risk score distribution and the vital status of patients in the high-risk and low-risk groups

According to the expression levels of EPDR1, we divided the cancer cases into 2 (By median), or 3 groups (By tertiles): low-expression, medium-expression, and high-expression and we explored the correlation of EPDR1 expression with the prognosis of patients with BLCA. Kaplan–Meier survival curves were conducted to draw the association between EPDR1 level and the survival outcomes of BLCA patients, mainly using the datasets of TCGA. We found a tendency that the BLCA patients with gradually ascending EPDR1 expression level would have a worse status, and the results showed that clustering of patients into two groups (Fig. 3a, *p* < 0.05, HR (low) = 0.621) or three groups (Fig. 3b, *p* < 0.05) in each cohort had significant differences in OS with BLCA. The AUC value of ROC analysis for the prognostic signature was 0.576, 0.568, and 0.559 for 1-year survival, 3-year survival, and 5-year survival, respectively (Fig. 3c). Notably, the number of surviving decreased, and cancer-related death increased with increasing risk scores, thus the group with high EPDR1 expression seemed as having a shorter OS compared to the low or medium expression-group (Fig. 3d).

### Building a predictive model

**Fig. 4:**
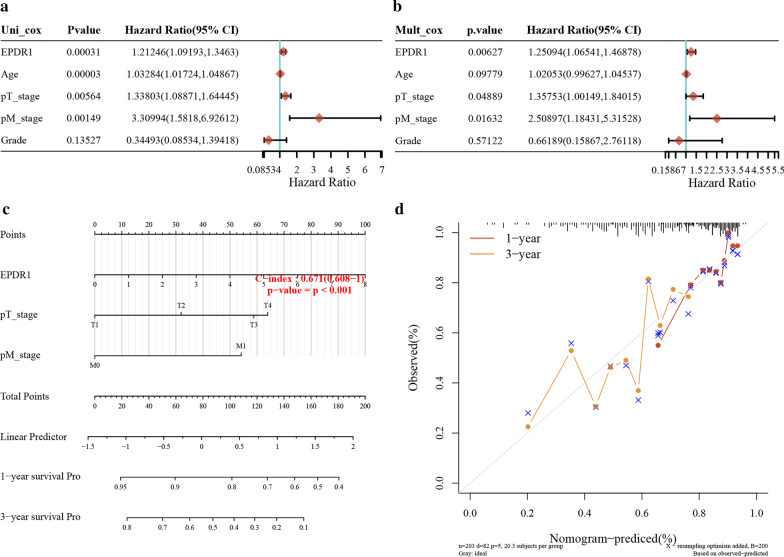
Predictive model. **a** Univariate analyses of clinicopathological characteristics and EPDR1 prognostic signature with overall survival in TCGA BLCA cohort; **b** multivariate analyses of clinicopathological characteristics and EPDR1 prognostic signature with overall survival in TCGA BLCA cohort; **c** Nomogram to predict the 1-year overall survival of BLCA patients. **b** Calibration curve for the overall survival nomogram model in the group. A dashed diagonal line represents the ideal nomogram, and the red line and yellow line represent the 1-y and 3-y observed nomograms

We resorted to a nomogram to building a predictive model that combines with some vital clinicopathological covariates. First of all, univariate and multivariate analyses of each factor were conducted to explore the HR in BLCA (Fig. 4a,b), and EPDR1 had a significant influence (HR = 1.2, *p* < 0.05) in the predictive model. Furthermore, we generated a nomogram to predict the 1-year and 3-year OS rates in the discovery group using the Cox regression algorithm (Fig. 4c). The predictors included EPDR1 expression, age of patients, pT-stage, pM-stage, and Grade, satisfying the criteria of *p* < 0.05 in risk assessment. Meanwhile, the plots for the 1-year and 3-year OS rates were predicted moderate compared with an ideal model in the entire cohort (Fig. 4d).

**Fig. 5:**
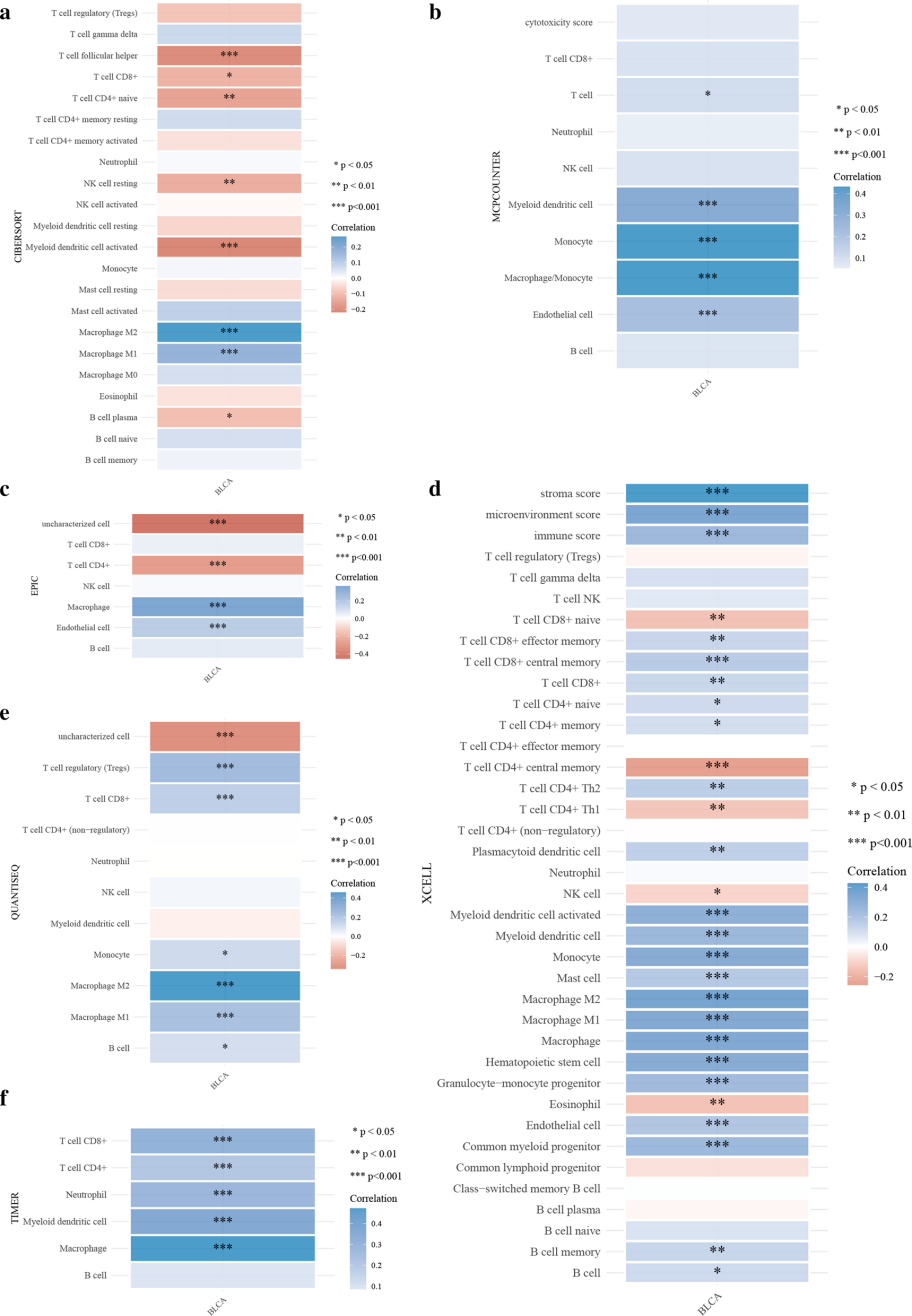
Relationship between EPDR1 expression and TIICs A-F: Immune score evaluation by TIMER, xCell, MCP-counter, CIBERSORT, EPIC, and quanTIseq: Different colors represent correlation coefficients, the horizontal and vertical coordinates represent genes, and different colors represent correlation coefficients (In the diagram, red represents positive correlation, blue represents negative correlation), and the darker the color represents the two stronger correlation. Asterisks represent levels of significance (*p < 0.05, **p < 0.01,***p < 0.001)

EPDR1 expression and tumor-infiltrating immune cells (TIICs). We explored the relationship between EPDR1 expression and immune cell infiltration in BLCA using an R software package that integrates six latest algorithms, including TIMER, xCell, MCP-counter, CIBERSORT, EPIC, and quanTIseq. All the outcomes (Fig. 5a–f) revealed that EPDR1 expression had a significant correlation with TIICs, including NK cell, CD8 + T cells, CD4 + T cells, Macrophages cells, Myeloid dendritic cells, Monocyte, Endothelial cells, and so on. Moreover, the Macrophage M1/M2 and Myeloid dendritic cells may have a strong correlation coefficient with EPDR1 expression.

### Enrichment analysis of EPDR1-related partners

**Fig. 6:**
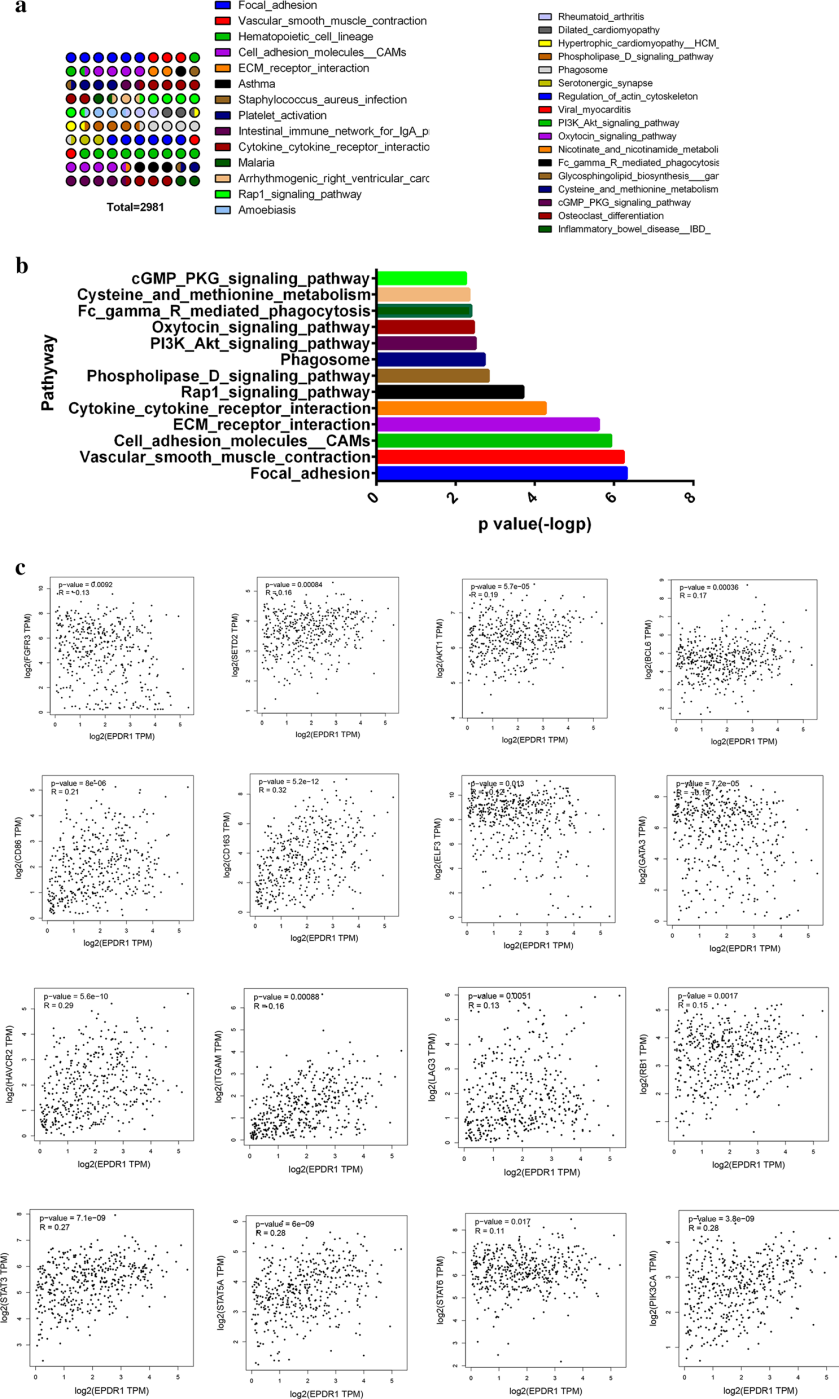
Enrichment analysis of EPDR1-related partners. **a** Go analysis of mRNAs highly significantly co-expressed with EPDR1; **b** Top pathway-related by KEGG; **c** gene expression correlation analysis plots

To gain a further investigation into the molecular mechanism and biological importance of the EPDR1 in BLCA, we attempted to screen out the EPDR1 expression-correlated genes and pathways by enrichment analyses. We used the GEPIA2 tool to combine BLCA data of TCGA and acquired the top 30 genes (Fig. 6a) that were highly co-expressed with EPDR1 based on Pearson correlation Coefficient. Moreover, enrichment of pathways by the KEGG database (Fig. 6b) showed that EPDR1 expression may have a great contributes to such ways of Cell adhesion molecules (CAMs), Chemokine signaling pathway, Cytokine cytokine receptor interaction, Focal adhesion, and so on. These results revealed that EPDR1 expression could have a widespread impact on the global transcriptome of BLCA tissues. Moreover, using methods including Pearson, Spearman, and Kendall, we conducted several pair-wise related gene expression correlation analyses of EPDR1 for given sets of TCGA and/or GTEx expression data. As the results showed that FGFR3, HIF-1, AKT1, PIK3CA, BCL6, CD86, CD163, ELF3, GATA3, HAVCR2, ITGAM, LAG3, STAT3, STAT5A, STAT6, and RB1 were prominently relatively expressed with EPDR1.

### MSI/ TMB analysis

**Fig. 7:**
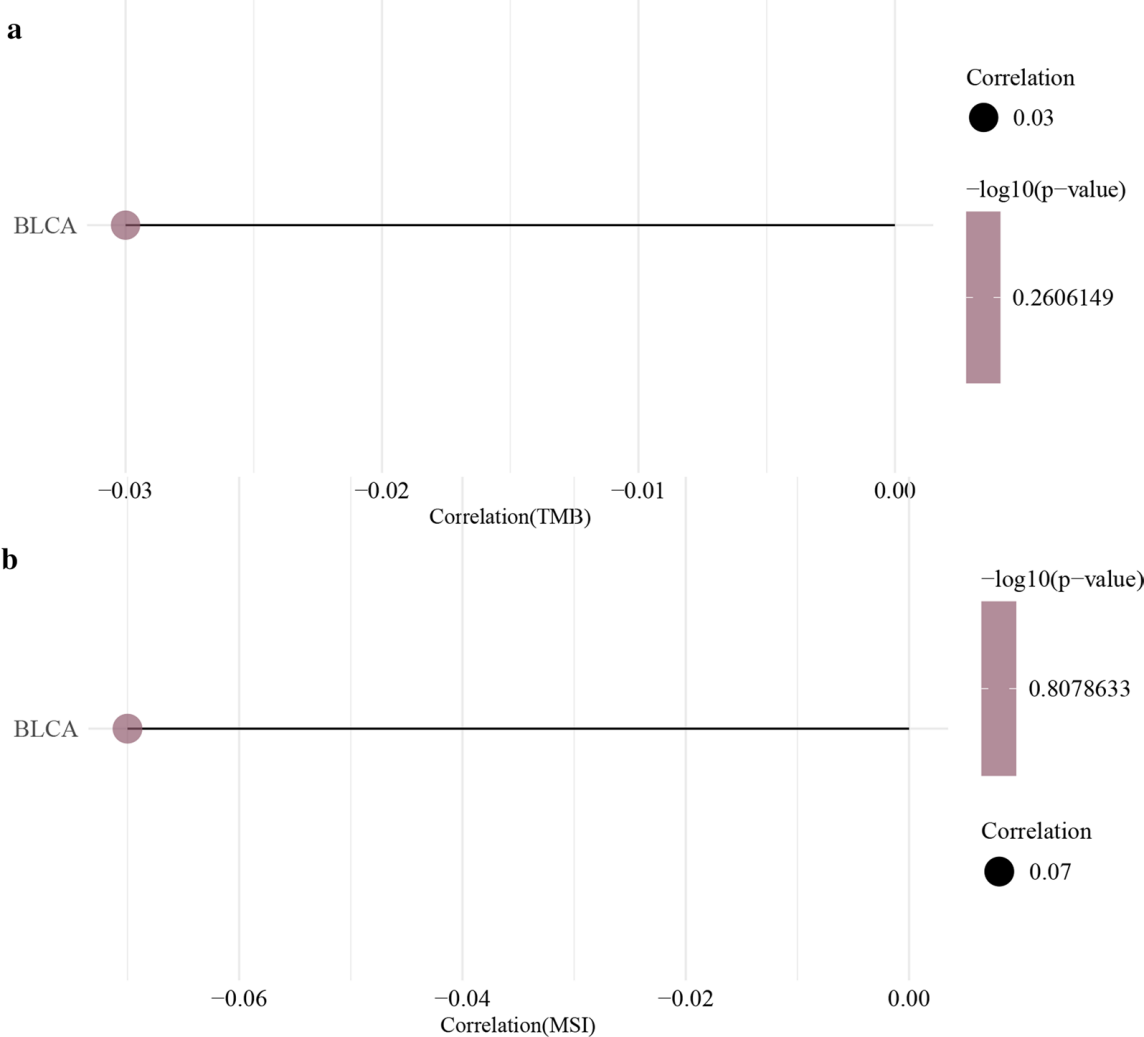
Spearman correlation analysis. **a** TMB and EPDR1; **b** MSI and EPDR1

To further explore the role of EPDR1 in the tumorigenesis of BLCA, we conducted a Spearman-Correlation analysis of MSI/TMB with EPDR1. However, it seemed that the correlation was pretty low and had no statistical significance (Fig. 7a,b).

## Discussion

Bladder cancer is a malignant urothelial (transitional cell) carcinoma and ranges from unaggressive and usually noninvasive tumors to aggressive and invasive tumors with high disease-specific mortality [14]. Advances in finding new biomarker and combining with the underlying biology of bladder cancer may fundamentally change how this disease is diagnosed and treated. EPDR1, which is similar to ependymins, may play a role in calcium-dependent cell adhesion. This protein is glycosylated, and the orthologous mouse protein is localized to the lysosome. In order to explore the potential functions of EPDR1 in BLCA, we proceeded with bioinformatics analysis by using publicly available data and hoped it will benefit future research related to BLCA.

In present studies, we explored the expression level of EPDR1 in BLCA, and consequently, we found that the BLCA patients with high EPDR1 expression are more likely to accompany with an advanced grade, stage, metastasis, and poor prognosis than those with low EPDR1 expression. To assess the prognostic value of EPDR1, we conducted an analysis of the OS rate for patients grouping different expressions of EPDR1 with the risk score, and interestingly, found that the P-value in all of the groups above was statistically significant. Compared with the low expression of ERDR1, the higher would significantly indicate worse OS in BLCA patients. In addition, we built a nomogram to predict individual 1- and 3-year overall survival rates, and the HR of EPDR1 has a statistical significance in the predicted model, no matter in univariate and multivariate analysis. Considering the above characters of EPDR1 in BLCA patients, our findings portended that EPDR1 would be a hazard for metastasis and horrible survival status, thus it could be a potential diagnostic and prognostic marker in BLCA.

To gain a further investigation into the biological role of EPDR1 in BLCA, we performed several functional analyses including the co-expressed genes, related TIICs, possible pathways and so on. Bladder cancer is known to be immunogenic and is responsive to immunotherapy [15], thus finding a biomarker in the mechanisms of immune evasion in BLCA that may contribute to weakening the tumor-induced immune escape and tolerance. Previous studies [16, 17] demonstrated that various immune cells counts in the tumor microenvironment (TME), such as Macrophages prime the pre-metastatic site, enable tumor cell extravasation and survival, and help to metastasis [17]; regulatory T cells (T regs) is to restrain chronic immune responses against viruses, tumors, and self-antigens, and contribute to tolerance [18]. We found that EPDR1 expression had a significant correlation with TIICs, including NK cell, CD8 + T cells, CD4 + T cells, Macrophages cells, Myeloid dendritic cells, Monocyte, Endothelial cells, and so on. Thus it suggested that EPDR1 expression with the infiltration levels of various immune cells may play a role in modulating cancer immunity. To probe the signaling pathways, we conducted the KEGG pathway and co-expressed genes analysis, and the results revealed that EPDR1 overexpression was involved in multiple signaling pathways, including PI3K/AKT pathway, Rap1 pathway, Cytokine receptor interaction, apoptosis, and so on. Considering these signaling pathways, we took a further step into the related genes and we demonstrated that those genes: FGFR3, HIF-1, AKT1, PIK3CA, BCL6, CD86, CD163, ELF3, GATA3, HAVCR2, ITGAM, LAG3, STAT3, STAT5A, STAT6, and RB1 had a significantly positive association with EPDR1. As previous studies [19, 20] had reported that above related signaling pathways and genes affected carcinogenesis and progression, we speculated that EPDR1 may participate in regulating progress and metastasis of BLCA.

In conclusion, our study indicated that EPDR1 could be detected as a novel biomarker with prognostic significance in BLCA patients. Meanwhile, EPDR1 expression was significantly related to different grades and metastasis, and it may play a role in modulating TIICs and affecting the potential molecular mechanism of the carcinogenesis and progression in BLCA. Therefore, we conclude that EPDR1 can be used as a biomarker or target for the diagnosis or treatment of BLCA.

## Data Availability

The data that support the findings of this study are available in multiple databases and repositories. These data were derived from the following resources available in the public domain: The Cancer Genome Atlas (TCGA) (https://cancergenome.nih.gov/); Gene Expression Profiling Interactive Analysis (GEPIA) database (http://gepia.cancer-pku.cn/); Gene-Expression-Omnibus-database (https://www.ncbi.nlm.nih.gov/gds); Kaplan–Meier-plotter (http://kmplot.com/analysis/); UCSC-Xena-project (http://xena.ucsc.edu/); UALCAN (http://ualcan.path.uab.edu); Linked Omics database (http://www.linkedomics.org/login.php); Tumor Immune Estimation Resource (TIMER) database (http://cistrome.org/TIMER/); CIBERSORT (https://cibersort.stanford.edu/); Human-Protein-Atlas (HPA) database (https://www.proteinatlas.org/); Clinical Proteomic Tumor Analysis Consortium (CPTAC) database (https://cptac-data-portal.georgetown.edu/cptacPublic/).
